# Structure Investigations of Islands with Atomic-Scale Boron–Carbon Bilayers in Heavily Boron-Doped Diamond Single Crystal: Origin of Stepwise Tensile Stress

**DOI:** 10.1186/s11671-021-03484-4

**Published:** 2021-02-08

**Authors:** S. N. Polyakov, V. N. Denisov, V. V. Denisov, S. I. Zholudev, A. A. Lomov, V. A. Moskalenko, S. P. Molchanov, S. Yu. Martyushov, S. A. Terentiev, V. D. Blank

**Affiliations:** 1 Technological Institute for Superhard and Novel Carbon Materials, Troitsk, Moscow, Russia 108840; 2 The PN Lebedev Physical Institute, Moscow, Russia 119991; 3 Institute of Spectroscopy, Russian Academy of Sciences, Troitsk, Moscow, Russia 108840; 4 Moscow Institute of Physics and Technology, Dolgoprudny, Moscow Region, Russia 141701; 5 Valiev Institute of Physics and Technology, Russian Academy of Sciences, Moscow, Russia 117218; 6 AV Topchiev Institute of Petrochemical Synthesis, Russian Academy of Sciences, Moscow, Russia 119991

**Keywords:** Boron-doped diamond, 2D nanoscale bilayers, Tensile stress, X-ray, Synchrotron nanobeam diffraction, Micro-Raman, Phase contrast in AFM

## Abstract

The detailed studies of the surface structure of synthetic boron-doped diamond single crystals using both conventional X-ray and synchrotron nano- and microbeam diffraction, as well as atomic force microscopy and micro-Raman spectroscopy, were carried out to clarify the recently discovered features in them. The arbitrary shaped islands towering above the (111) diamond surface are formed at the final stage of the crystal growth. Their lateral dimensions are from several to tens of microns and their height is from 0.5 to 3 μm. The highly nonequilibrium conditions of crystal growth enhance the boron solubility and, therefore, lead to an increase of the boron concentrations in the islands on the surface up to 10^22^ cm^−3^, eventually generating significant stresses in them. The stress in the islands is found to be the volumetric tensile stress. This conclusion is based on the stepwise shift of the diamond Raman peak toward lower frequencies from 1328 to 1300 cm^−1^ in various islands and on the observation of the shift of three low-intensity reflections at 2-theta Bragg angles of 41.468°, 41.940° and 42.413° in the X-ray diffractogram to the left relative to the (111) diamond reflection at 2theta = 43.93°. We believe that the origin of the stepwise tensile stress is a discrete change in the distances between boron–carbon layers with the step of 6.18 Å. This supposition explains also the stepwise (step of 5 cm^−1^) behavior of the diamond Raman peak shift. Two approaches based on the combined application of Raman scattering and X-ray diffraction data allowed determination of the values of stresses both in lateral and normal directions. The maximum tensile stress in the direction normal to the surface reaches 63.6 GPa, close to the fracture limit of diamond, equal to 90 GPa along the [111] crystallographic direction. The presented experimental results unambiguously confirm our previously proposed structural model of the boron-doped diamond containing two-dimensional boron–carbon nanosheets and bilayers.

## Introduction

The unique properties of diamond as the ultra-wide band gap semiconductor make it indispensable in high-power and RF electronics, optoelectronics, quantum information and extreme-environment applications. Two main advances may be indicated in the record of the synthesis of semiconductor diamonds by high-pressure high-temperature (HPHT) technique [1]. The first advance was associated with the development of growth technology for the large-size high-quality single-crystal diamonds [2–4]. The second advance was elaboration of the technique of effective doping of diamond with boron (B) and phosphorus (P) in a wide range of concentrations [5–7]. Fabrication of diamond with high B and P concentration is complicated by the high formation energies of substitutional B, P in the diamond lattice. High formation energy implies low equilibrium dopant solubility. The boron solubility can be enhanced with the tensile stresses, as theoretically predicted in [8]. Articles [9, 10] demonstrate that the biaxial tensile stress leads to a significant increase of the boron solubility in silicon. Very high boron solubility in diamond was achieved under highly nonequilibrium conditions of growth [11].

We discovered recently a formation of a two-dimensional (2D) layer structure in the boron-doped diamond (BDD) [5]. The B atoms are mainly incorporated into nanosheets and bilayers, enhancing the boron solubility in the diamond lattice. Since superconductivity was observed only on the BDD surface [12], there is a need for a more detailed study of the 2D layered structure on the as-grown surface. Superconductivity in the bulk of the BDD single crystal was not observed because the boron concentration was low (~ 0.13 at.%). However, the transition to the superconducting state was obtained at boron concentration of 2 at. % with critical temperature (*T*_c_) equal to 2 K [13]. Moreover, the B concentration of 8 × 10^21^ cm^−3^ (4.55 at.%) can be achieved in CVD films providing *T*_c_ of 8.3 K [14]. The boron concentrations on the BDD surface are more than one order of magnitude higher than in its bulk, and the reason for this is still to be determined. To clarify it we studied the difference between bulk and surface structure of large-size single crystals. The presence of a deep acceptor level of 0.37 eV in BDD also limits the boron solubility. We found earlier a new shallow acceptor level of 0.037 eV, formed at the B concentrations above 4 × 10^18^ cm^−3^ (0.0023 at.%) in BDD single crystals, which can also increase the bulk solubility of boron in them.

We observed shifts of the diamond peak position in Raman spectra obtained from different points on {111} faces of the BDD from 1328 to 1300 cm^−1^, indicating high tensile stresses. Similar diamond peak shifts observed in CVD polycrystalline BDD films were also explained by the residual stress in them [15–17]. The shifts of the diamond phonon line from 1328 to 1300 cm^−1^ showed a surprising stepwise behavior with a step of 5 cm^−1^, never before detected in BDD [5]. Such discrete shifts are inherent to materials with a 2D layered structure and were observed in Raman spectra of graphene and hexagonal boron nitride [18, 19]. We found that the shifts of the diamond peak in different areas of the surface had different values, and, hence, different magnitudes of residual stress. More suitable nondestructive methods with high spatial resolution should be used to quantify the magnitudes of these stresses and to determine the cause of the stepwise shift of the phonon peak. In this paper, we report on the results of detailed studies of the as-grown {111} surfaces of a BDD single crystal using micro-Raman spectroscopy, conventional X-ray and synchrotron nanobeam diffraction, X-ray reflectivity and phase contrast in tapping mode of atomic force microscopy.

## Methods

### Synthesis of the Boron-Doped Diamond Single Crystals

The BDD single crystals were grown by the HPHT method at high pressure of 5.5 GPa and high temperature of 1440 °C in the “toroid” type cell [2]. The Fe–Al–C alloy with the element ratio 91:5:4 wt%, respectively, was used as the solvent metal. The aluminum was added to the solvent as the nitrogen getter. High purity graphite (99.9995%) was used as the carbon source and amorphous boron powder was applied as the doping component. Synthetic diamond crystals with cross-sectional size of ~ 0.5 mm and (100) surface orientation were used as seeds. The temperature in the high-pressure cell was measured with the accuracy of 2 °C by Pt6%Rh–Pt30%Rh thermocouple. The temperature gradient between the carbon source and the seed crystal was ~ 30 °C.

The BDD single crystals with boron concentration of 0.13 at.% in the bulk were cut by a technological laser into plates with as-grown {111} faces for detailed studies. The surfaces opposite to the as-grown one were polished to remove the graphitized layer remaining after cutting [20].

### Experimental Techniques

The Empyrean X-ray diffractometer (PANalytical, Netherlands) equipped with a PIXcel^3D^ detector providing high sensitivity and high linearity range of 0–6.5 × 10^9^ counts per second was used for registration of the diffraction patterns of boron-doped diamond plates with an X-ray beam irradiating the entire surfaces of these. The nanobeam diffraction mapping was carried out at the ID01 and ID13 beamlines of the European Synchrotron Radiation Facility (ESRF, Grenoble, France). The synchrotron X-ray beams with transverse size of 2 × 2 µm^2^ and 180 × 180 nm^2^, respectively, were utilized for local analysis. The SmartLab Rigaku (Japan) diffractometer was applied for acquisition of the specular X-ray reflectivity (XRR) curves. The Renishaw inVia confocal Raman microscope with an argon ion laser operated at the excitation wavelength of 514.5 nm was used for Raman spectra measurements with spectral resolution of 1 cm^−1^. The spatial resolution of ~ 1 μm and the probing depth of ~ 2 μm were achieved with the confocal Raman microscope. The surface topography and the atomic composition of the as-grown {111} BDD faces were measured with the SolverBio atomic force microscope (NT-MDT, Russia), equipped with the silicon nitride probe with the curvature radius less than 10 nm.

## Results and Discussion

**Fig. 1:**
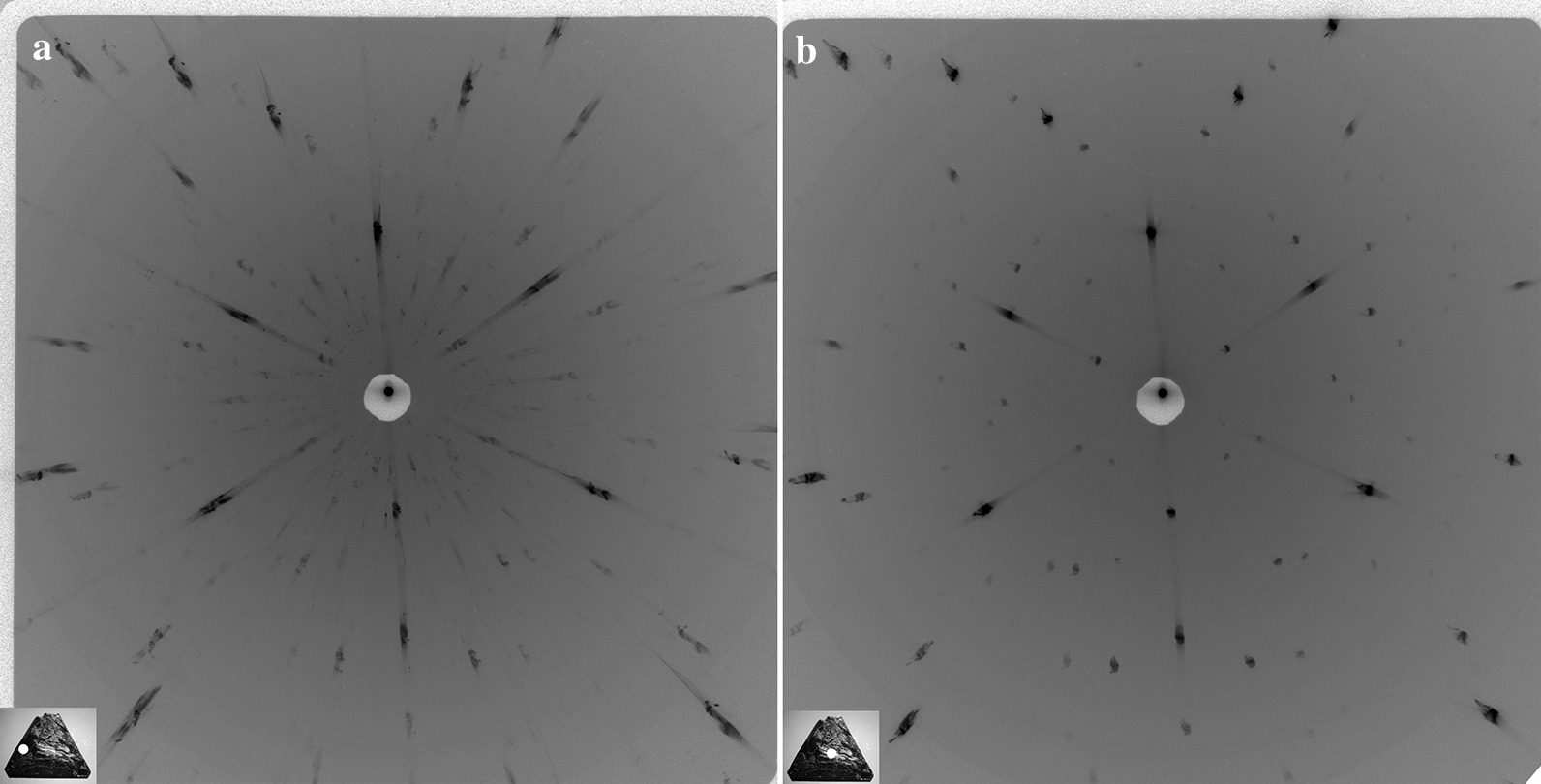
X-ray transmission Laue patterns obtained from: **a** peripheral area of the BDD plate and **b** central area of the BDD plate. The radial streaks in Laue patterns are caused by the diamond crystal lattice distortion

The photograph of the as-grown {111} face of the studied BDD plate with the thickness of 0.5 mm is shown in Additional file 1: Fig. S1. The polished surface opposite to the as-grown one was used to obtain detailed experimental data on the bulk properties of the BDD as a reference for the data from the as-grown surface. The first part of the studies was the examination of the BDD plate with the Laue method. The 9-kW rotating anode X-ray generator with a tungsten target providing the ideal bremsstrahlung spectrum was used for the lauegram registration. The X-ray beam of 0.5 mm diameter illuminating the as-grown (111) surface of the BDD plate was formed with a double pinhole collimator. A coarse mapping was performed in the transmission geometry to record the X-ray Laue patterns. Twelve lauegrams obtained from central and peripheral areas of the plate are shown in Additional file 1: Fig. S2. Two lauegrams illustrate the presence of extra Laue spots in the peripheral areas of the BDD plate (Fig. 1a) and their absence in the central areas (Fig. 1b). The extra Laue spots indicate the presence of islands with 2D layered structure in this area. The appearance of radial streaks (asterism) observed in the lauegram in Fig. 1a reveals significant distortion of the diamond lattice.

**Fig. 2:**
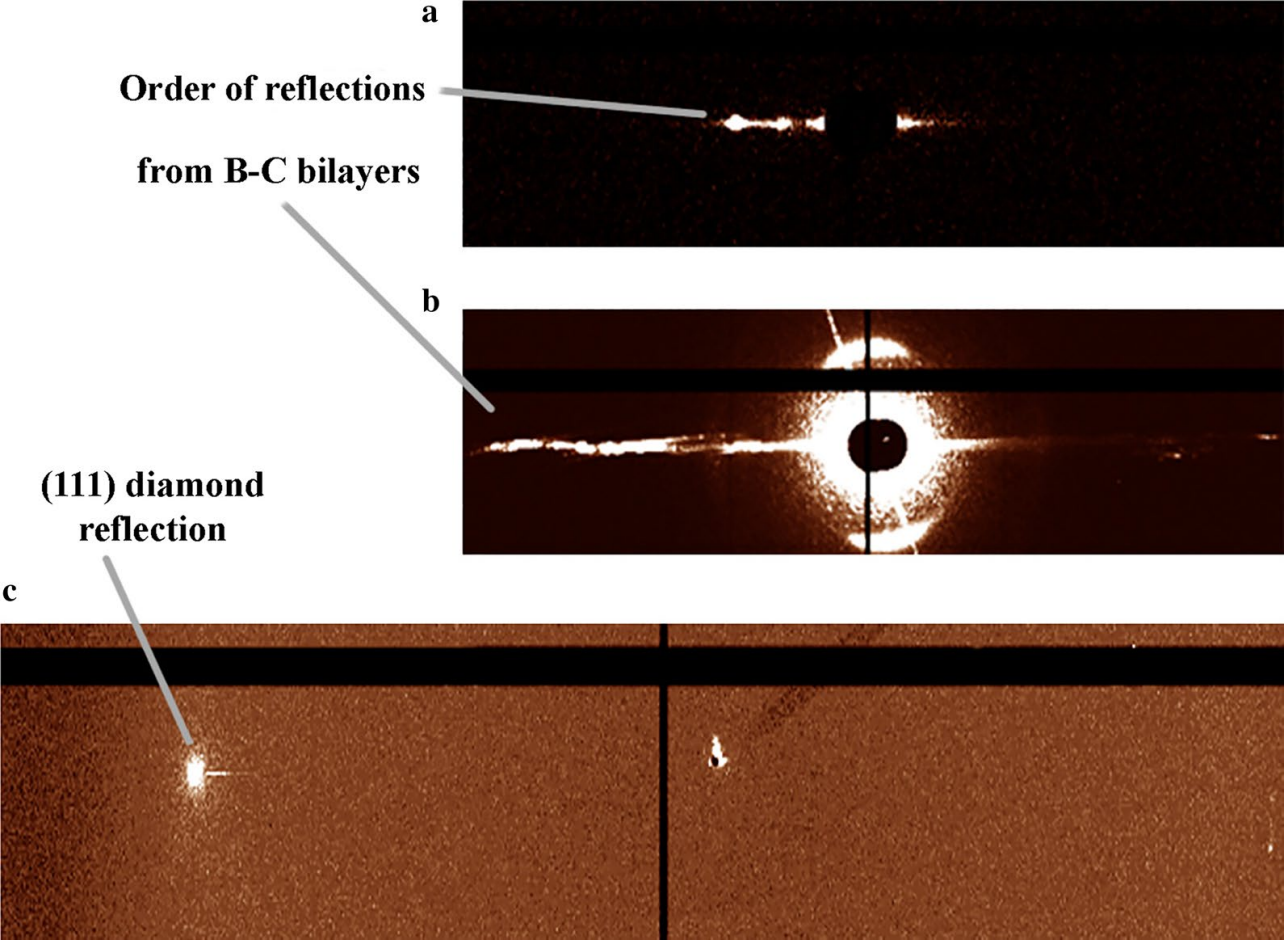
X-ray nanobeam diffraction patterns obtained from a local area of BDD plate: **a** 2D image of the diffraction pattern, **b** the same diffraction pattern in another intensity scale and **c** X-ray diffraction pattern, recorded with a lower intensity of the primary X-ray beam, allowing the observation of the high-intensity (111) diamond reflection

To determine the lateral size of the areas with the 2D layered structure more precisely, the synchrotron nanobeam diffraction studies were carried out at the ID13 nanofocus beamline of the ESRF. The energy of the monochromatic X-ray nanobeam used for local analysis was equal to 14.9 keV (*λ* = 0.853 Å) with the size of 180 × 180 nm^2^. The photograph of the area with dimensions of 140 × 200 µm^2^ corresponding to the part of the sample surface marked with a circle in Fig. 1a is shown in Additional file 1: Fig. S3. This area contained the maximum number of extra Laue spots. The 2D diffractograms at the ID13 beamline were recorded in the (x,y) field-of-view with a step of 600 nm. To analyze the entire area of 140 × 200 µm^2^, it was divided into 70 sections. Mapping with the focused monochromatic nanobeam in the reflection mode was carried out for each section separately to simplify the data processing afterward. A total number of 43,750 diffractograms obtained from 70 sections (625 diffractograms for each section) was analyzed. The lateral sizes of islands were estimated based on the fact that the diffraction pattern remained unchanged within specific section. Additional file 1: Fig. S4 shows the set of X-ray diffractograms taken from two different sections of the BDD plate surface demonstrating the presence of islands with different sizes. We have established that the islands had an arbitrary shape and their lateral dimensions ranged from several microns to tens of microns. The 2D diffractograms from the local area with the 2D layered structure are presented in Fig. 2. The superlattice reflections are clearly observed in the angular range between the primary beam and the (111) diamond reflection and can be unambiguously identified as orders of reflection from layers with a longer period compared to the interplanar spacings of the host diamond structure. Hence, the analysis of the data obtained with the Laue method and with the synchrotron nanobeam diffraction allows to draw a conclusion that islands with the 2D layered structure were formed on the BDD surface.

**Fig. 3:**
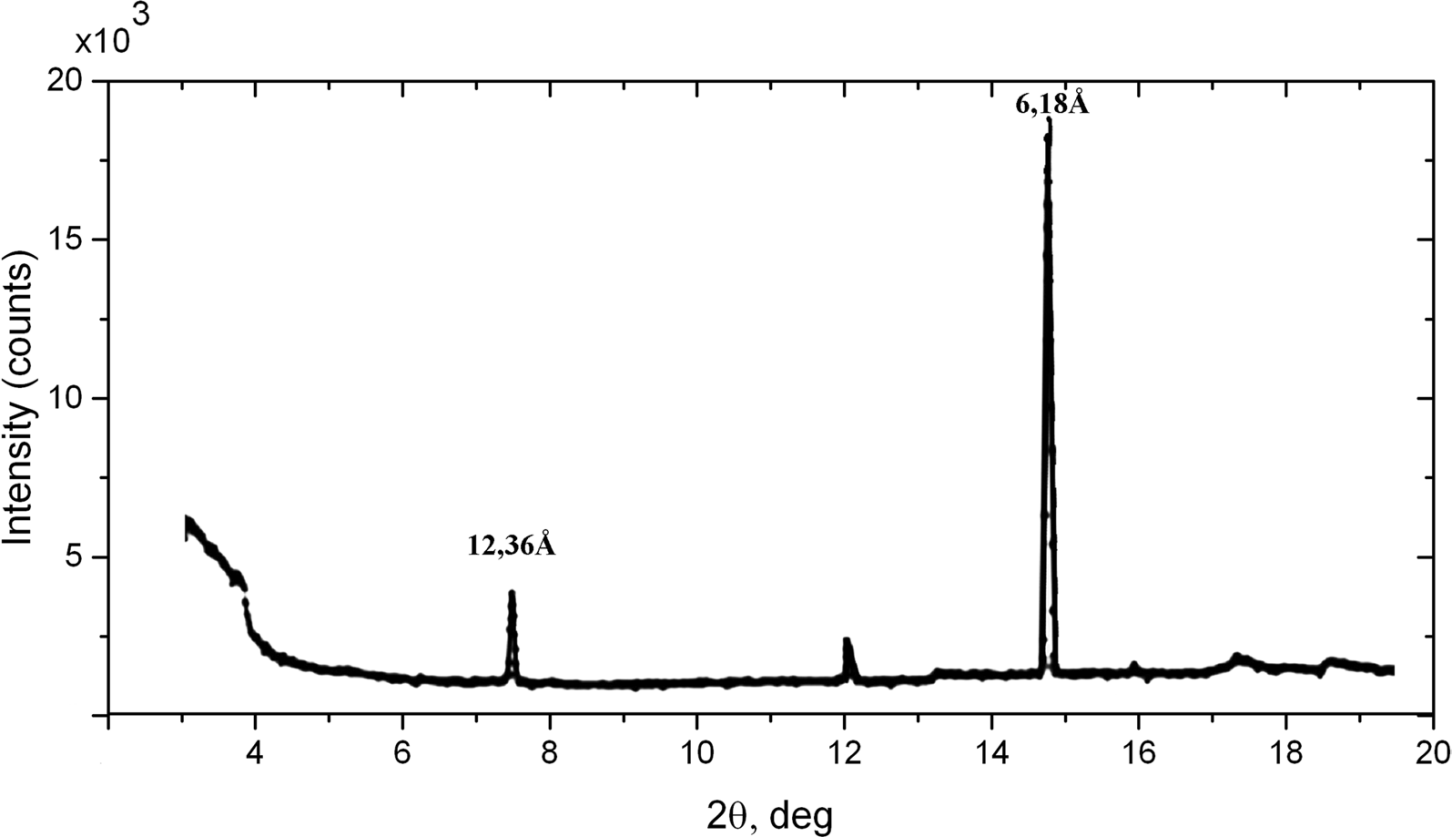
X-ray synchrotron diffraction pattern (ID01, ESRF) taken from the middle part of the narrow BDD plate. The most intense reflections correspond to the distances between boron–carbon layers 12.36 and 6.18 Å. Low-intensity reflections originate from islands with other periods (not indexed). In particular, the peak at 2*θ* = 12.2° can be assigned as fifth order from islands with period equal to ~ 37.08 Å

Consequently, the relations should be established between the boron concentration in the individual islands and their structural parameters. In order to determine the periods between the B-C layers in the islands on the surface of the BDD single crystal, we applied a softer X-ray synchrotron radiation. Experiments were carried out at the ID01 microdiffraction imaging beamline of the ESRF. The X-ray microbeam with the energy of 7.8 keV (*λ* = 1.597 Å) was used to obtain the diffraction pattern. The diffraction pattern was recorded on the Maxipix photon-counting pixel detector with 55 µm pixel size [21] with slits set to 2 × 2 µm^2^. In order to decrease the effect of the surface vertical inhomogeneity, narrow plate with dimensions of 0.5 (width) × 0.5 (thickness) × 4 (length) mm^3^ containing superlattice reflections was cut from the BDD plate (Additional file 1: Fig. S1b). Since the angle of incidence of the X-ray beam on a sample is small, the diffraction pattern is produced only by the subsurface volume. Figure 3 shows the X-ray diffraction pattern taken from the middle area of the narrow plate in Additional file 1: Fig. S1b. The superlattice reflections are clearly observed. The most intense X-ray reflection at 2*θ* = 14.85° corresponds to the smallest possible period of 6.18 Å. We also succeeded in observation of the superlattice reflections with the period of 12.36 Å (2*θ* = 7.41°). The superlattice reflections with longer periods could not be detected due to the presence of the high-intensity “tail” from the primary beam.

**Fig. 4:**
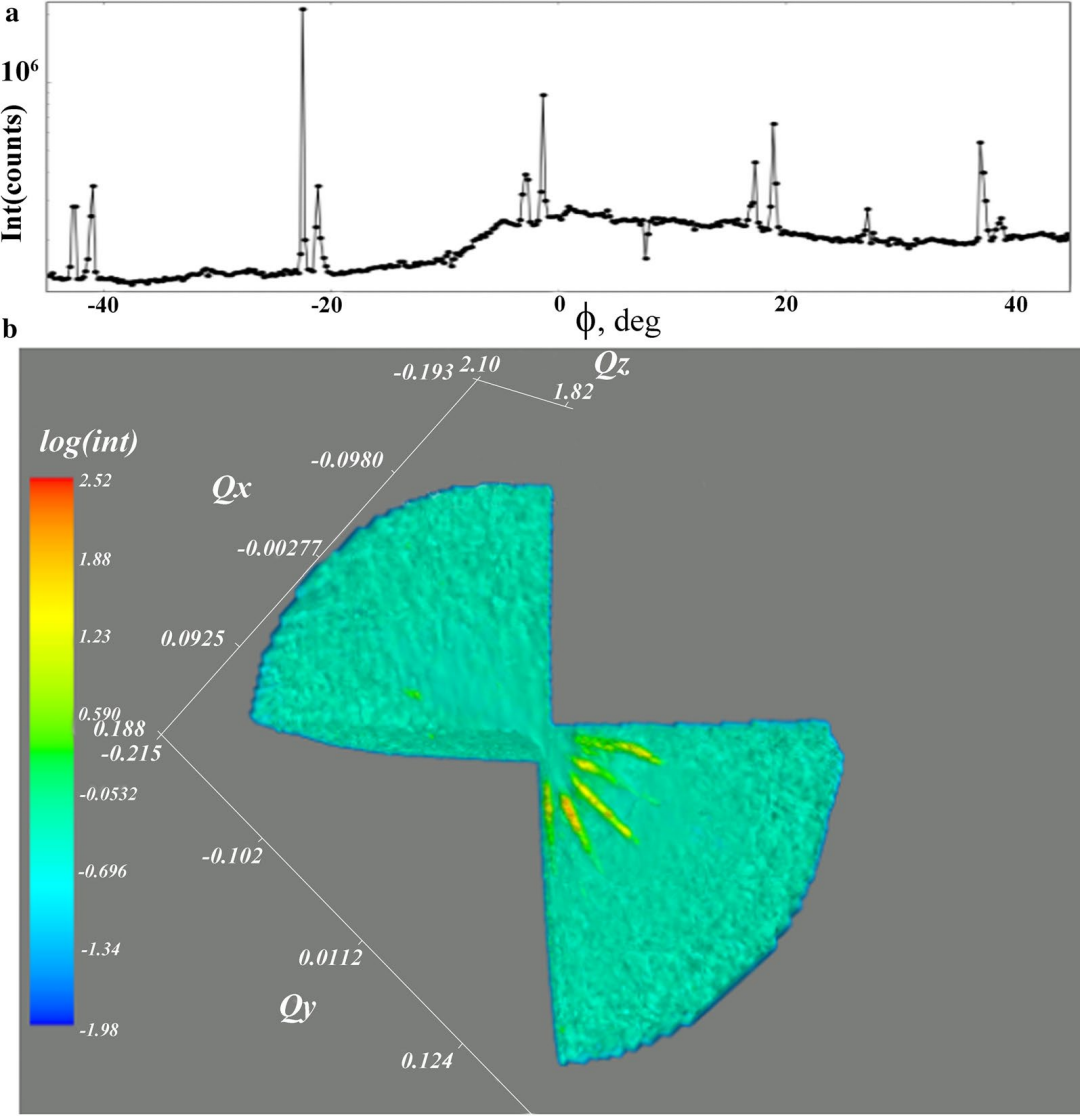
**a** Synchrotron X-ray *ϕ*-scan diffraction pattern of the narrow BDD plate. **b** Reciprocal space representation of wave vectors from 2D layered structure with the period of 6.18 Å (yellow streaks)

We conclude that the observation of the reflections with the smallest possible period indicates the presence of islands on the surface in which the boron concentration reaches the maximum value according to the model of 2D layered structure [5]. The highest boron concentration in BDD results in the maximum stress of diamond lattice. The observation of the maximal Raman diamond peak shift to the value of 1300 cm^−1^ confirms this fact. We suppose that the lower intensity of reflection from the islands with the period of 12.36 Å is due to a smaller number of layers in them or only a part of surface of the island with such a period was involved in diffraction because of the small transverse size of the incoming X-ray beam. To obtain additional information about the structure of the islands with the smallest period, the reciprocal space measurements in the vicinity of the superlattice reflection at 2*θ* = 14.85° were carried out. The Maxipix detector was set to the stated 2*θ* position, and scanning of the sample around the *ϕ* axis normal to the sample surface was performed from − 45° to 45°. The results of *ϕ*-scanning are shown in Fig. 4a. Five doubled reflections, separated by 20°, can be seen in the figure. The origin of double reflections on the *ϕ*-scan curve (see Fig. 4a) can be explained using the model of the BDD structure proposed in [5]. Additional file 1: Fig. S5a shows the distribution of boron (blue) and carbon (gray) atoms in the ($\bar{1}10$) plane. Since the B–C bonds (1.6 Å) are longer than the C–C bonds (1.54 Å), the boron atoms are shifted toward each other along the broken chemical bonds in the [111] direction (marked by strokes). The displacement of boron atoms leads to the formation of crystallographic planes, with interplane distances between which are incommensurate with the distances in the basic structure (see Additional file 1: Fig. S5a). Additional file 1: Fig. S5b shows an isometric illustration of the BDD structure. It demonstrates directions of wave vectors in 3D space whose length is incommensurate (red) and commensurate (black) with the vectors of the periodic host structure. Thus, this clarifies the appearance of double reflections on the *ϕ*-scan curves. The combination of incommensurate and commensurate wave vectors leads to the formation of a number of wave vectors, the lengths and directions of which do not coincide with that of the vectors of the host structure, explaining the presence of extra spots in the Laue patterns and five double reflections on *φ*-scan curve (Fig. 4b). We believe that the same structural features are inherent in the islands with other periods.

**Fig. 5:**
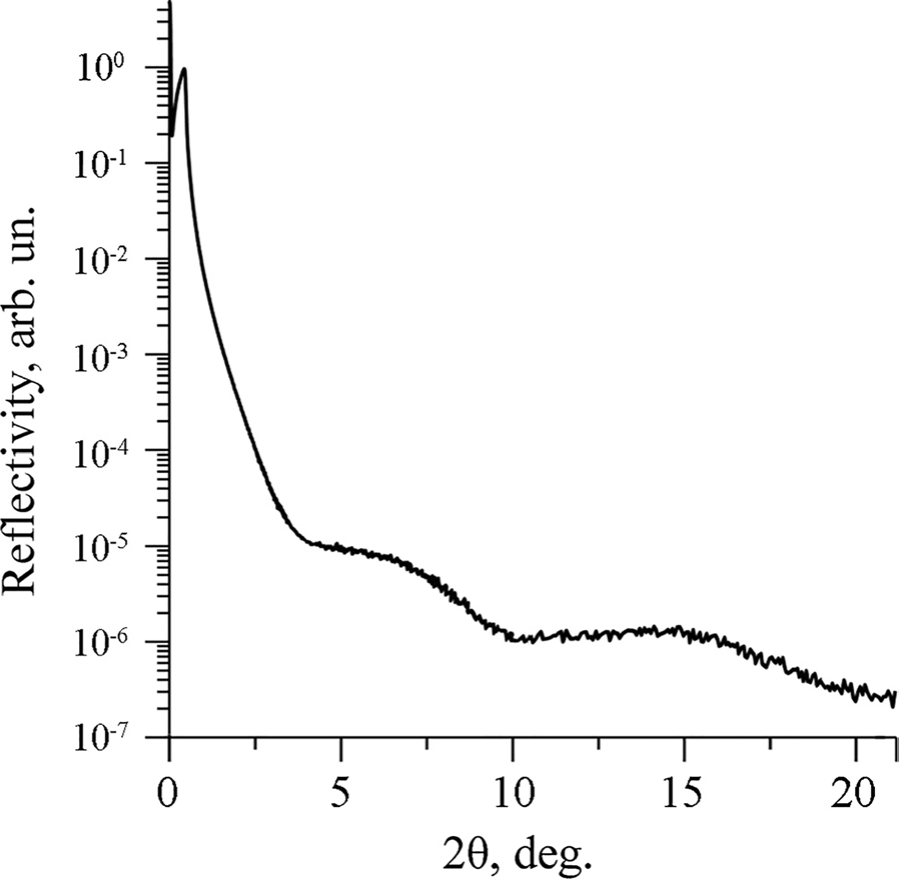
Experimental X-ray reflectivity curve of the polished BDD plate

The XRR technique is usually implemented to determine the structural parameters of islands on the as-grown BDD surface, such as the spacing between layers and the number of layers. Since the as-grown surface of the BDD plate shows an inhomogeneous topography (see Additional file 1: Fig. S1a), application of this technique is hardly possible. However, this method can be used to define these structural parameters in the BDD bulk. In order to retrieve this information, we experimentally studied the polished surface of the BDD plate opposite to the as-grown one. Conclusions about the structural parameters of the 2D layers in the bulk are based on the comparison of the experimental specular reflection curves with the theoretical ones. The IMD software for modeling and analysis of a multilayer film was used to simulate the theoretical curves [22]. The specular curve demonstrates the orders of the reflections from the layers and the oscillations between them caused by the interference of the X-ray waves reflected from the B-C layers. The thickness of the boron–carbon layers, the number of layers, the X-ray wavelength, the 2*θ* angular range and the scan step were entered into the IMD software as parameters for the theoretical curve simulation. The theoretical and the experimental specular reflectivity curves are shown in Additional file 1: Fig. S6 and Fig. 5, respectively.

Two broad peaks in the experimental specular reflection curve at 2*θ* ≈ 7 and 15° are the orders of reflection from the extremely small-size islands, also called nanosheets. The absence of oscillations is probably associated with small lateral dimensions and different periods of oscillations produced by individual nanosheets. The average lateral size of the nanosheets estimated from the peaks broadening is equal to ~ 2 nm.

**Fig. 6:**
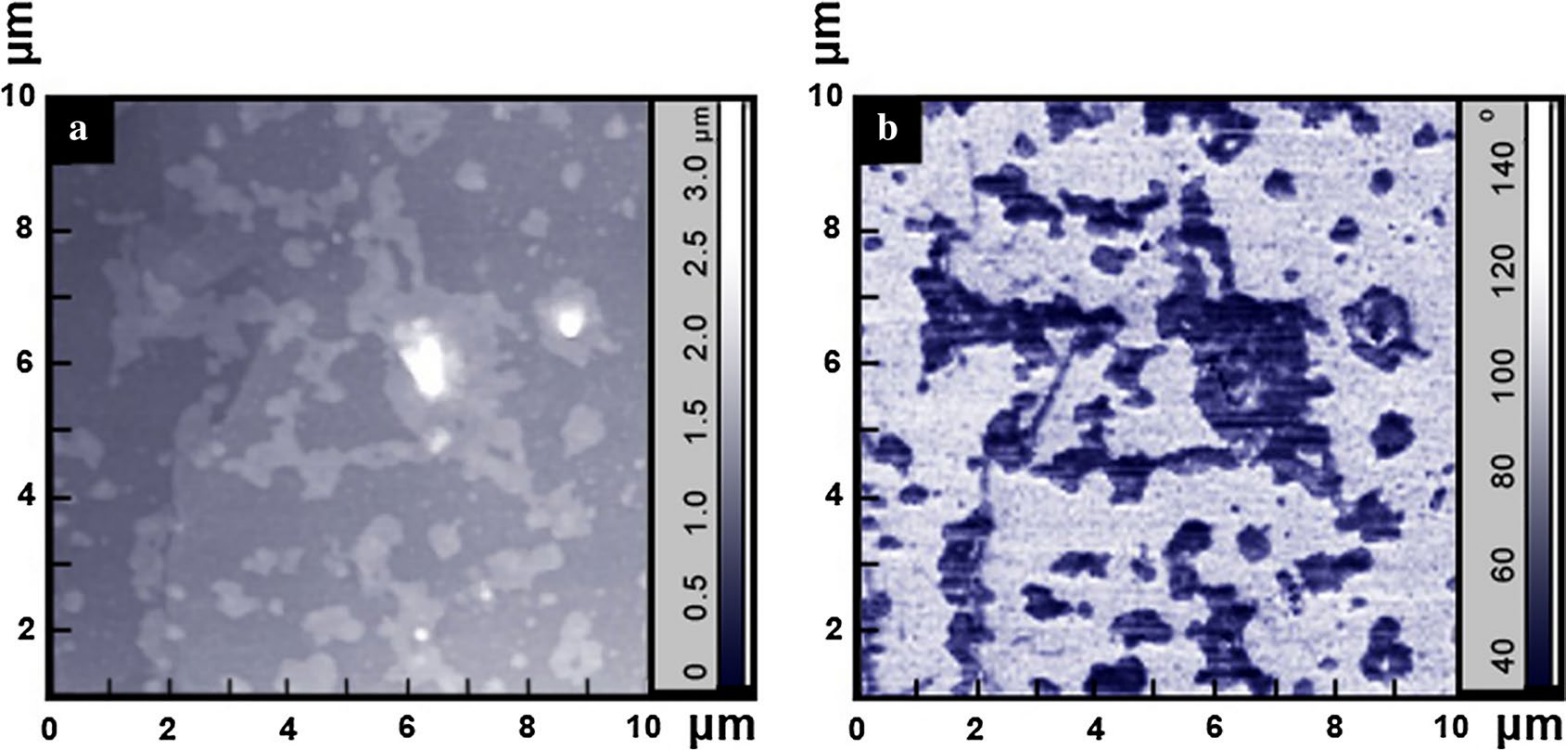
**a** AFM image of the BDD obtained in surface-relief height scanning mode. **b** Phase contrast image in tapping-mode AFM of the same BDD area

The surface topography is usually studied using atomic force microscopy. Two basic modes can be applied for surface analysis. The first is the standard mode for determining the height of the surface structures. The second is the phase contrast mode, which provides information about the difference in atomic composition of various surface areas. As a result, the phase contrast mode can be used to determine the lateral dimensions of islands with different concentrations of boron in them. We used atomic force microscopy (AFM) to determine the height of islands. Figure 6a shows the 10 × 10 μm^2^ AFM image of the BDD obtained in the surface topography height scanning mode. The arbitrary shaped islands with lateral sizes from fractions of microns to tens of microns are clearly visible and their heights vary from 0.5 to 3 μm. The phase contrast image in the tapping mode of the same BDD region is presented in Fig. 6b. The observed dark and bright areas are associated with the phase shifts in areas of different atomic composition. As can be seen in Fig. 6b, the bright areas are related to the host diamond and the dark ones to the islands with higher boron concentration. A comparison of the images Fig. 6a, b allows to draw a conclusion that the dark areas are the islands towering above the host diamond surface. Since the lateral sizes of the islands obtained with the X-ray nanobeam diffraction mapping are in an agreement with those provided by the AFM observations, we conclude that the towering dark areas are the islands with the 2D layered structure.

**Fig. 7:**
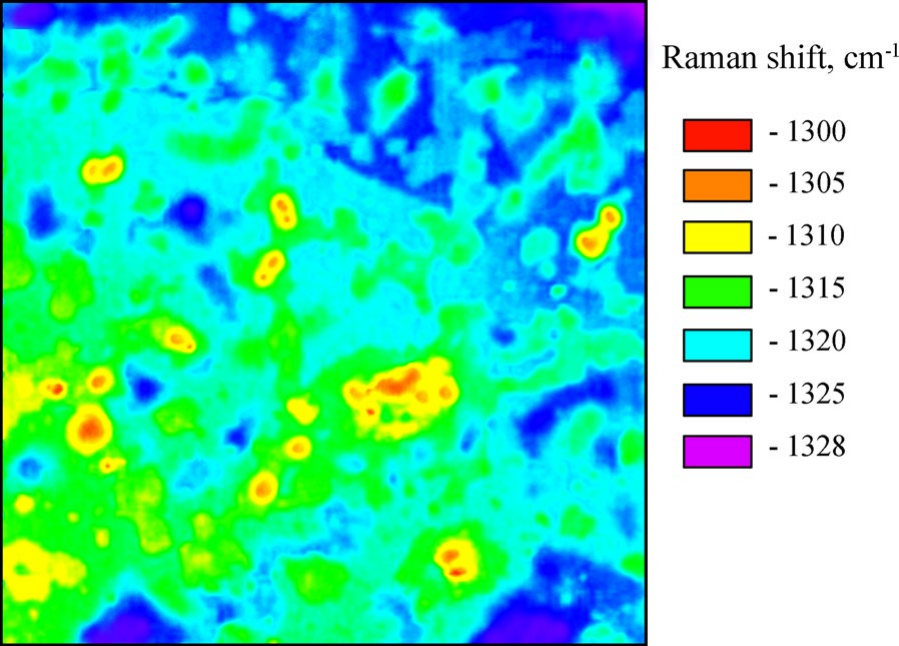
Image of fine Raman mapping of the 150 × 150 μm^2^ surface area of the narrow BDD plate. Colors indicate the diamond Raman peak position at different surface areas

In this regard, the strain in the islands and its dependence on the boron concentration should be determined. Another important task is to clarify the origin of the stepwise behavior of the shift of the Raman peak of diamond. For this purpose, the Raman mapping of the central part of the narrow BDD plate was performed. Due to the strong resonant absorption at a laser wavelength of 514.5 nm, the Raman scattering probes the surface layers within the penetration depth of several tens of nanometers. The 3 mW excitation laser beam focused into a spot of ~ 1 μm diameter was used. At this power, the laser heating of the diamond surface and islands with the 2D layered structure in the focused spot was negligible. The characteristic Raman spectra from different areas of the as-grown (111) surface of the BDD plate (coarse Raman mapping) are shown in Additional file 1: Fig. S7. The fine Raman mapping (step of 1.5 μm and an exposure time of 3 s at every point) of the 150 × 150 μm^2^ surface area of the narrow BDD plate marked by the white square in Additional file 1: Fig. S1b is shown in Fig. 7. Fitting with a Lorentz function was applied to the Raman spectra to create Raman mapping images for the diamond peak position. The automatic focus tracking mode was used to compensate the irregular height of the surface.

The total number of 10,000 Raman spectra was analyzed. The Raman mapping analysis shows that the position of the diamond phonon peak was constant within areas on the as-grown surface, marked by different colors, but changed from one area to another. As shown in Fig. 7, the position of this peak varies stepwise from 1328 to 1300 cm^−1^ with a step of ~ 5 cm^−1^. The diamond phonon peak at 1328 cm^−1^ marked with the violet color in Fig. 7 coincides with that in the Raman spectrum of the BDD bulk. Histogram, shown in Additional file 1: Fig. S8, exhibits the area ratio of islands with different boron concentrations. Different boron concentrations generate a different stress leading to different diamond peak shift.

The investigations of the BDD surface structure by the local methods given above demonstrated the formation of arbitrary shaped islands, towering above the host diamond surface. The islands have the lateral dimensions from several to tens of microns with the heights from 0.5 to 3 μm. The first reason for the formation of the islands is the growth of BDD in highly nonequilibrium conditions at the final stage of crystallization after switching off the HPHT apparatus. The growth of islands under such conditions leads to the increase of boron solubility and the boron concentration rises up to 10^22^ cm^−3^ in them. The second reason refers to the presence of horizontal and vertical boron concentration gradients at the interface between the growth environment and the surface of the growing crystal. It was found that the boron concentrations in the islands are different, which creates different stresses in each of them. The reason for the appearance of residual stresses in the islands is the incorporation of boron atoms into the cubic diamond lattice at doping. Since the covalent radius of the dopant boron atom (0.88 Å) is greater than that of the carbon (0.77 Å) that leads to an increase in the lattice constant of cubic diamond unit cell [23]. Since each island towering above the diamond host surface can be considered as a separate microcrystal, volumetric residual stress should be generated in them. We emphasize that the structure of the boron-doped diamond films grown by CVD method differs from that of the BDD single crystals grown by HPHT. Boron atoms in these films are homogeneously distributed over large areas, which create a biaxial residual stress equilibrated throughout the whole film. This residual stress can be classified as Type I and refers to macro-residual stresses that develop on a scale larger than the crystallite size of the materials [24]. On the other hand, the residual stress in islands (microcrystals) can be considered as the superposition of Type II and Type III often called micro-residual stress. The Type II micro-residual stresses operate at the microcrystal-size level. The Type III micro-residual stresses are generated on the atomic level due to an incorporation of boron pairs in the diamond unit cell. We believe that an increase in the micro-residual stress in the islands is associated with the distances between B–C bilayers. It should be noted the islands with 2D layered structure are coherently conjugated with the host diamond lattice according to the structural model proposed in [5]. This implies that there is no sharp interface between bulk diamond and islands, and therefore no substantial mismatch strain.

**Fig. 8:**
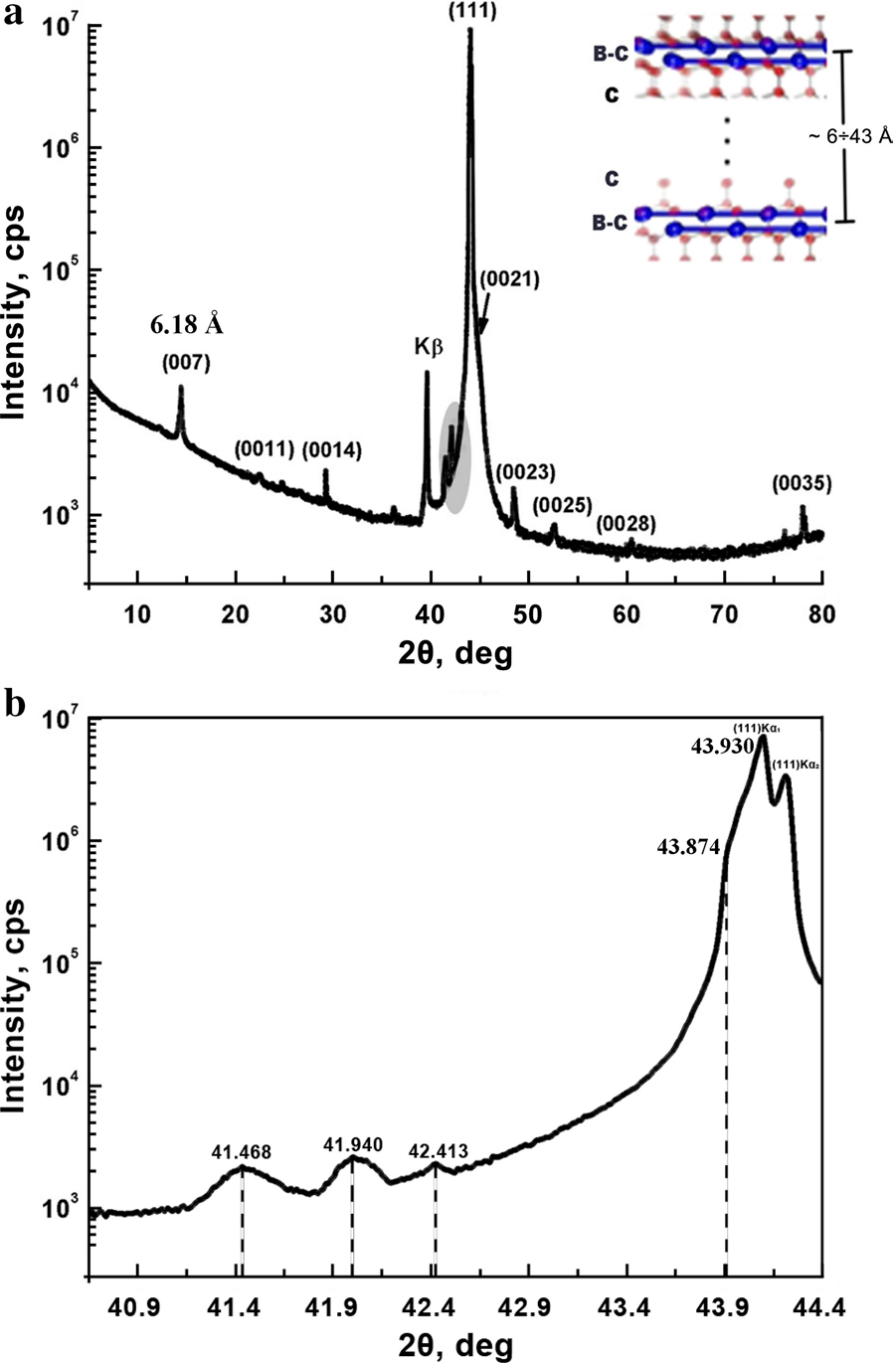
**a** X-ray diffraction pattern (θ/2θ-scan) of the single-crystal BDD plate with the (111) surface orientation. The inset (top right) shows the layout of boron–carbon layers in a cubic diamond matrix with distances between them from ~ 6 to 43 Å. **b** Part of the diffractogram **a** containing the area marked with gray color in an enlarged scale

X-ray diffraction is the most suitable method for measuring elastic deformations of crystalline materials. It should be noted that X-ray Sin^2^*ψ* method is normally used for stress determination in the polycrystalline materials only and cannot be applied for stress measurements in single crystals. Bragg–Brentano geometry is more suitable for determination of elastic deformation in the direction normal to the BDD surface both in different islands with 2D bilayers on the BDD surface and in the host diamond because the incoming X-ray beam illuminates the whole sample surface and penetrates into the narrow plate to the ~ 200 μm depth. X-ray diffraction patterns were recorded using the Empyrean X-ray diffractometer equipped with the PIXcel3D detector and the Bragg-Brentano HD optical module for improved data quality. The parameters of diffraction patterns acquisition allowed simultaneous observation of both weak reflections from islands and the strong (111) diamond reflection with intensity ~ 4 orders of magnitude higher. Figure 8a shows the X-ray diffraction pattern (*θ*/2*θ*-scan) of the BDD plate with the (111) surface orientation.

The strong (111) reflection from diamond and the weak reflections representing the reflections from the islands with the 2D layered structure are observed in the X-ray diffraction pattern. The most intense of the weak reflections at 2*θ* = 14.3° is attributed to diffraction on islands with the minimum distance between the B-C layers of 6.18 Å. It was surprising to observe three weak separate reflections at angles 2*θ* equal to 41.468°, 41.940° and 42.413° with intervals of Δ2*θ* ≅ 0.470° in the vicinity of the (111) reflection (Fig. 8b). These peaks cannot be related to some orders of reflection and their appearance should be clarified. We believe that their presence is due to high stepwise deformation of the diamond lattice in the islands. This conclusion is based on the fact that the islands with minimal possible distances between B-C layers are present on the surface. Indeed, orders of reflections with periods of 6.18 and 12.36 Å were observed in X-ray diffraction pattern obtained from the central area of the narrow plate at the ID01 synchrotron beamline (Fig. 6). The Raman mapping analysis of the same areas demonstrated the presence of islands with the Raman diamond phonon peak also stepwise shifted to the values of 1300, 1305 and 1310 cm^−1^. Thereby, we conclude that the origin of the stepwise tensile strain of the diamond lattice in the islands is due to the discrete change of spacing between B–C layers.

The volumetric (triaxial) residual stress is characterized by the principal stresses *σ*_*x*_, *σ*_*y*_, *σ*_*z*_, which are determined using the generalized Hooke's law. Taking into account the transverse and the longitudinal expansions in the directions of the principal axes, we obtain the strains by means of the following expressions [25]:

1 $$\begin{gathered} \varepsilon_{1} = \frac{1}{E}[\sigma_{1} - \nu (\sigma_{2} + \sigma_{3} )] \hfill \\ \varepsilon_{2} = \frac{1}{E}[\sigma_{2} - \nu (\sigma_{3} + \sigma_{1} )] \hfill \\ \varepsilon_{3} = \frac{1}{E}[\sigma_{3} - \nu (\sigma_{1} + \sigma_{2} )], \hfill \\ \end{gathered}$$

where *ε*_1_, *ε*_2_, *ε*_3_ are the strains along the principal axes, *E* is the Young modulus, *ν* is the Poisson ratio, *σ*_*x*_ = *σ*_1_, *σ*_*y*_ = *σ*_2_, *σ*_*z*_ = *σ*_3_ are the stresses along the principal axes.

There are two approaches to estimate *σ*_1_, *σ*_2_, *σ*_3_. The first approach is based on the combining of data obtained from the X-ray diffraction and the Raman scattering. X-ray diffraction provides measurements of the elastic deformation in the transverse direction, while Raman scattering allows it to be determined in the longitudinal direction at certain assumptions. There is a well-known equation for the dependence of the biaxial stress on the phonon diamond peak shift in the case of *σ*_3_ = 0 [17]:

2 $$\sigma_{||} = \sigma_{1} + \sigma_{2} = - 1.49\,{\text{GPa/cm}}^{ - 1} \times (\omega_{{\text{s}}} - \omega_{0} ),$$

where *ω*_s_ is the phonon diamond peak position shifted under stress, and *ω*_0_ corresponds to the position of the phonon peak centered at 1328 cm^−1^ in the BDD bulk. The validity of using this formula for triaxial stress is a question of contention. We suppose this equation can be used in the thin layer approximation taking into account the significant resonant absorption of laser radiation (514.5 nm) in B–C bilayers with metallic conductivity. This supposition is supported by the experimental fact that the integral intensities of 480 and 1230 cm^−1^ broad bands remain constant while the intensity of the phonon diamond peak decreases significantly (see Additional file 1: Fig. S7). The strain in the normal direction *σ* is obtained from the following equation:

3 $$\sigma_{3} = \varepsilon_{3} \times E + \nu \times (\sigma_{1} + \sigma_{2} ),$$

where *σ*_3_ = *σ*_⟂_ and *ε*_3_ is determined by expression:

4 $$\varepsilon_{3} = \Delta \theta \times ctg\theta^{\prime } ,$$

where Δ*θ* *=* *θ*_0_ − *θ*′, *θ*_0_ is the position of the unstrained diamond (111) Bragg reflection, corresponding to the maximum on the *θ*/2*θ* curve (2*θ*_0_ = 43.93°, Fig. 8), *θ*′ corresponds to the maximum of the three weak separate reflections at 2*θ* angles equal to 41.468°, 41.940° and 42.413°.

Taking into account the values of the Young modulus *E*_∥_ = 1164 GPa and the Poisson ratio *ν*_∥_ = 0.0791 [26], the numerical values of *σ*_∥_ and *σ*_⟂_ can be calculated using Eqs. (2), (3) and (4). The calculation results are presented in Table 1.

**Table 1:** Values of *σ*_∥_ and *σ*_⟂_ calculated according to Raman spectroscopy and X-ray diffraction data

Phonon line position *ω* (cm^−1^)	Shift *ω*_S_ − *ω*_0_ (cm^−1^)	Δ*θ=θ*_0_ − *θ*' (°)	*σ*_∥_ (GPa)	*σ*_⟂_ (GPa)
1310	18	0.727	26.8	38.5
1305	23	0.963	34.3	50.5
1300	28	1.200	41.7	63.6

As can be seen from the table, the maximum normal stress *σ*_⟂_ in the islands with minimum period of 6.18 Å is equal to 63.6 GPa, close to the diamond fracture limit at 90 GPa calculated theoretically for the given crystallographic direction [27].

The second approach is based on the hydrostatic diamond lattice expansion in islands. In this case *σ* *=* *σ*_1_ *=* *σ*_2_ *=* *σ*_3_ can be estimated from the equation:

5 $$\sigma = \varepsilon \times E/(1 - \nu ),$$

where *E*/(1 − *ν*) = 1264 GPa [26], *ε* = Δ*θ* × ctg*θ*′, *ε* = *ε*_1_ = *ε*_2_ = *ε*_3_. Strain *ε* is determined for each reflection centered at 41.468°, 41.940° and 42.413° on the *θ*/2*θ*-scan diffractogram (Fig. 8). The calculation results for hydrostatic diamond lattice expansion are presented in Table 2.

**Table 2:** Values of *σ* calculated according to the X-ray diffraction data

Position of Bragg reflection *θ*′ (°)	Δ*θ=θ*_0_ − *θ* ' (°)	*σ* (GPa)
20.734	0.727	42.35
20.970	0.963	55.40
21.2065	1.200	68.19

Calculation data based on two approaches showed that the values of *σ* and *σ*_⟂_ differ by approximately 10%. The values of *σ*_∥_ and *σ*_⟂_ estimated by the first approach differ by about one-and-a-half times.

The first approach looks more realistic taking in account 2D layered structure of islands. As far as we know, the anisotropic stress is a characteristic feature of 2D structures [28]. The question of the real values of the elasticity constants in view of the complex islands’ structure remains open. Determination of the quantitative values of Young modulus and Poisson ratio taking into account all real factors such as high values of stress in islands and their complex crystalline structure is a rather difficult task.

We have also determined the stress *σ* in the BDD bulk knowing the 2*θ*_Bragg_ position of the unstrained diamond (111) reflection at 2*θ*_0_ = 43.93° and the measured left-shift of reflection (2*θ*′ = 43.874°, Fig. 8b) caused by the stress in the bulk of host diamond. The estimated stress in the bulk is *σ* = *σ*_⟂_ = *σ*_∥_ = 1.528 GPa, assuming hydrostatic diamond lattice expansion using the relation (5) at Δ*θ =* *θ*_0_ − *θ*′ = 0.028°. This result correlates well with the data obtained by the synchrotron X-ray microbeam diffraction using the monochromatic X-ray beam with the energy of 7.8 keV (λ = 1.597 Å) where the (111) reflection splitting was also observed (see Additional file 1: Fig. S9). The calculated value *σ* of 1.528 GPa makes it possible to refine the coefficient of hydrostatic shift rate k = (*ω*_s_ − *ω*_0_)/*σ*. In this equation, the diamond phonon peak positions at *ω*_0_ = 1332 cm^−1^ and *ω*_s_ = 1328 cm^−1^ correspond to the undoped diamond and the diamond doped with the boron with concentration of 2 × 10^20^ cm^−3^, respectively. The refined value of the coefficient *k* = 2.68 cm^−1^/GPa is in agreement with the values obtained by other authors [29].

## Conclusions

In summary, we have studied the structure of islands with atomic-scale B-C bilayers on the BDD surface using various experimental techniques, namely synchrotron X-ray nano- and microbeam diffraction, conventional X-ray diffraction, atomic force microscopy and micro-Raman spectroscopy, to explain the characteristic features we observed in them. The arbitrary shaped islands, towering above the diamond surface, have lateral dimensions from several to tens of microns and heights from 0.5 to 3 μm. They are formed at the final stage of the BDD growth at highly nonequilibrium conditions, increasing the boron concentration in the islands up to ~ 10^22^ cm^−3^ that eventually generates significant stresses. It has been experimentally established that this stress is triaxial and tensile. This conclusion is based on the facts that the diamond Raman peaks are shifted toward lower frequencies down to 1300 cm^−1^ and the X-ray diffraction to the left from the strong (111) diamond reflection contains three low-intensity reflections at 2*Θ* Bragg angles of 41.468°, 41.940° and 42.413°. We believe that these three Bragg reflections are caused by the discrete change in tensile strain determined by the distance between boron–carbon layers with the step of 6.18 Å. This supposition explains the stepped behavior of the shift of the diamond Raman peak with the 5-cm^−1^ step. Two approaches based on the use of Raman scattering and X-ray diffraction data made it possible to estimate quantitatively the values of the stresses in lateral and normal directions. The calculated stress value reaches 63.6 GPa in the islands with the maximum boron concentration, close to the theoretically calculated fracture limit of diamond in the ˂111˃ direction. On the other hand, the experimentally determined tensile stress in the BDD bulk, equal to 1.528 GPa, is much smaller. The reliability of the previously proposed model of the 2D layered structure was confirmed by the experimental data obtained using a combination of multiple techniques.

## Supplementary Information

**Additional file 1.** Supplementary information for Structure investigations of Islands with Atomic-Scale Boron–Carbon Bilayers in Heavily Boron-Doped Diamond Single Crystal: Origin of Stepwise Tensile Stress.

## Data Availability

All data generated and analyzed during this study are included in this article.

## Abbreviations

BDD: Boron doped diamond
B–C: Boron–carbon
2D: Two-dimensional
HPHT: High pressure high temperature
AFM: Atomic force microscopy
