# Supplementary material for: Structure Investigations of Islands with Atomic-Scale Boron–Carbon Bilayers in Heavily Boron-Doped Diamond Single Crystal: Origin of Stepwise Tensile Stress
Source: Nanoscale Res Lett. 2021 Feb 8;16:25. doi: 10.1186/s11671-021-03484-4 (PMC7870744; doi:10.1186/s11671-021-03484-4)
Supplement: Supplementary file 1 — Additional file 1. Supplementary information for Structure investigations of Islands with Atomic-Scale Boron–Carbon Bilayers in Heavily Boron-Doped Diamond Single Crystal: Origin of Stepwise Tensile Stress. [file 11671_2021_3484_MOESM1_ESM.docx]

**Supplementary information**

**Structure Investigations of Islands with Atomic-scale Boron-Carbon Bilayers in Heavily Boron-doped Diamond Single Crystal: Origin of Stepwise Tensile Stress**

**SN Polyakov^1,2,6*^, VN Denisov^1,3,4*^, VV Denisov^1^, SI Zholudev^1^, AA Lomov^5^, VA Moskalenko^4^, SP Molchanov^6^, SYu Martyushov^1^, SA Terentiev^1^, VD Blank^1,4^**

* Corresponding authors: SN Polyakov [spolyakov@phys.msu.ru](mailto:spolyakov@phys.msu.ru)

VN Denisov denisovvn@tisnum.ru

^1^ Technological Institute for Superhard and Novel Carbon Materials, Moscow, Troitsk, 108840, Russia.

^2^ The PN Lebedev Physical Institute, Moscow 119991, Russia.

^3^ Institute of Spectroscopy, Russian Academy of Sciences, Moscow, Troitsk, 108840, Russia.

^4^ Moscow Institute of Physics and Technology, Dolgoprudny, Moscow Region, 141701, Russia.

^5^ Valiev Institute of Physics and Technology, Russian Academy of Sciences, Moscow, 117218, Russia

^6^ AV Topchiev Institute of Petrochemical Synthesis, Russian Academy of Sciences, Moscow, 119991, Russia.

**Sample preparation**

The photo of the as-grown {111} face with a complex surface topography of the boron-doped diamond (BDD) plate is shown in Fig. S1. Surfaces with such topography were only formed in BDD single crystals with the maximum possible boron (B) concentration in the bulk ~ 0.13 at. % when ~ 5.4 wt. % of amorphous boron powder was added to the growth environment. For detailed study of both as-grown surface and opposite polished one the 1 mm thick plate was laser cut from HPHT-grown BDD single crystal with tetrahedral habitus.

**
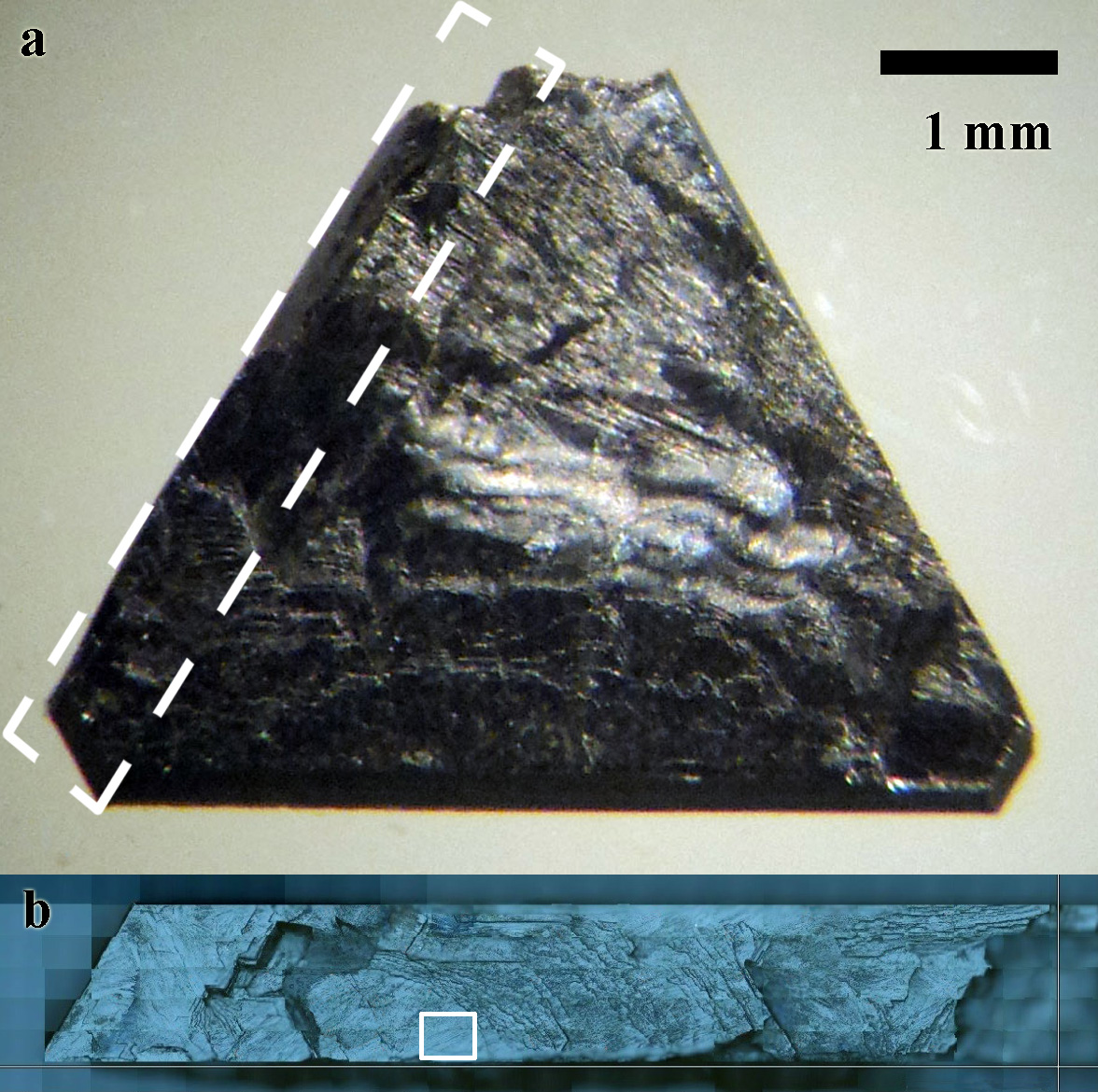
**

**Fig. S1. a** The photo of the as-grown {111} face of BDD plate. **b** The narrow BDD plate cut by laser from triangular plate (marked with white color in **a).** The area in white rectangle was used for mapping with X-ray nanobeam diffraction and Raman scattering.


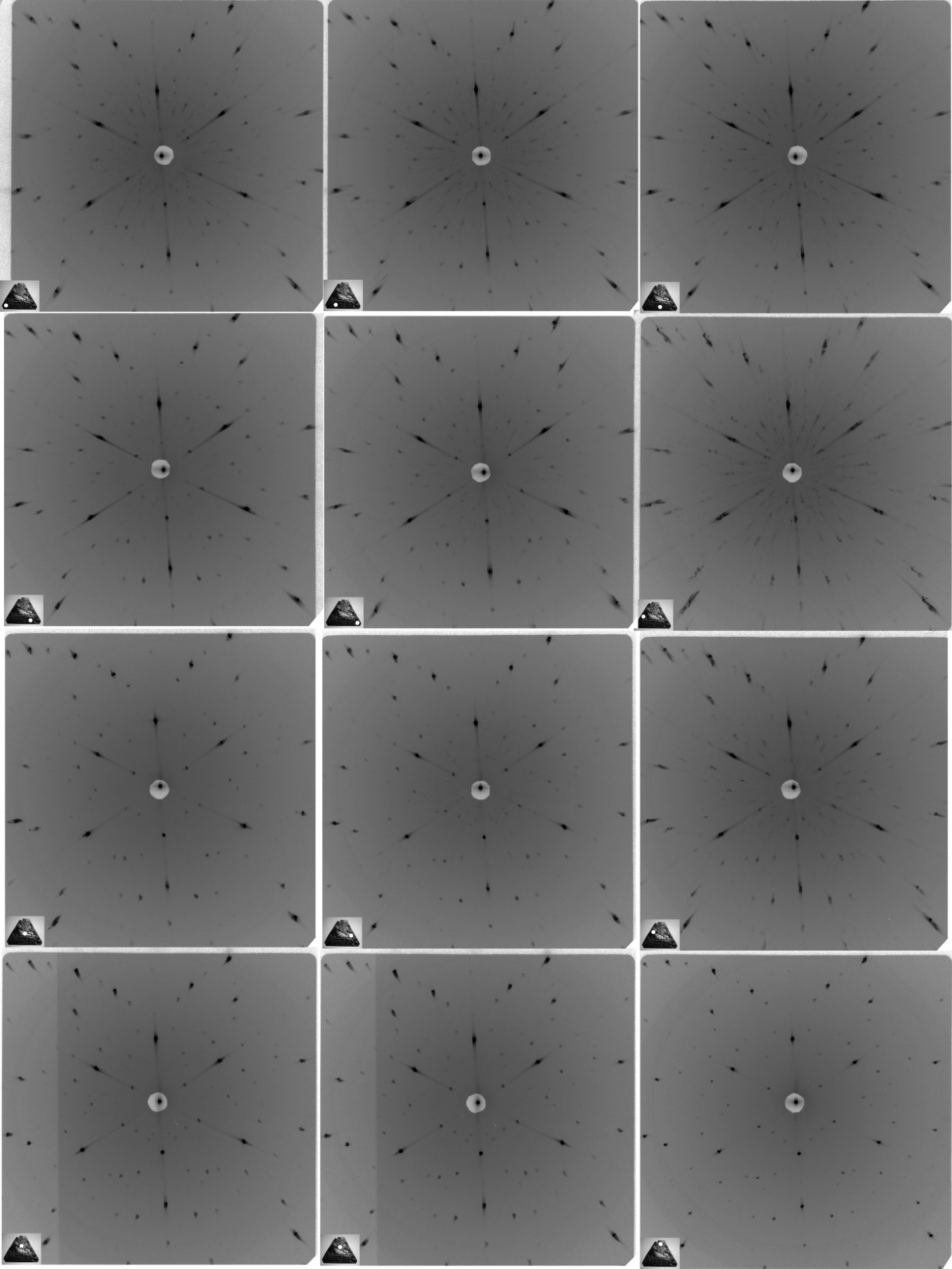


**Fig. S2**. Set of twelve lauegrams obtained from central and peripheral areas of the triangle BDD plate using X-ray beam with diameter of 0.5 mm marked by white spot.

**
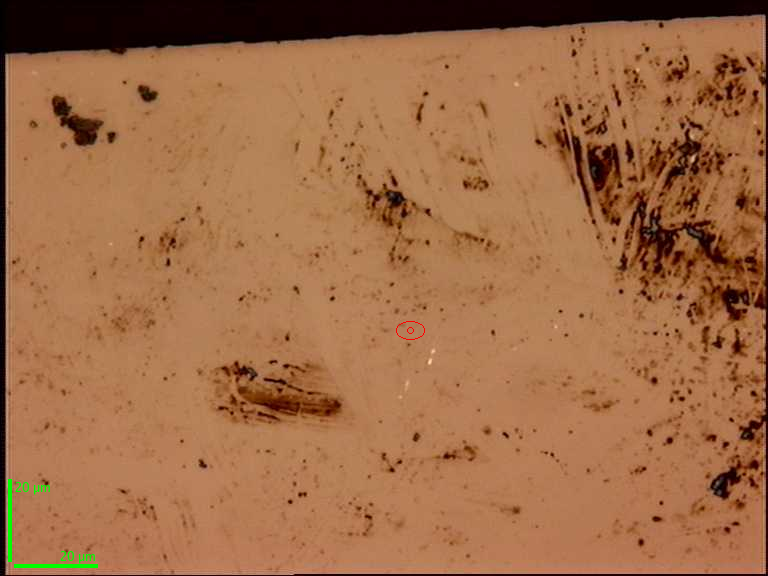
**

**Fig. S3.** Photo of the plate surface area of 140x200 μm, which was divided into 70 sections with a size of 20x20 μm. Each such area was used for mapping using nano-focused X-ray beam of 180x180 nm^2^ with the step of 600 nm.


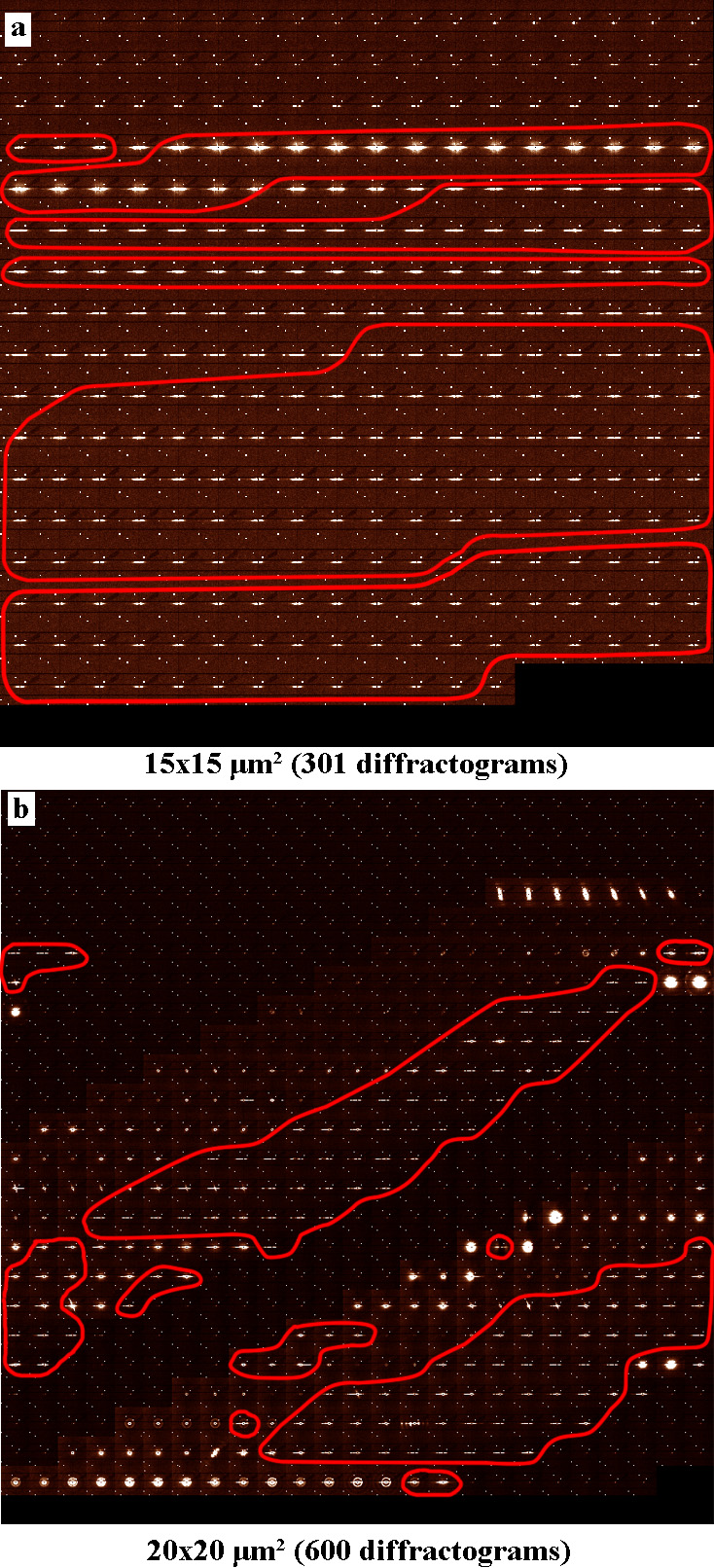


**Fig. S4.**  Sets of X-ray 2D-diffractograms taken from two different areas of the plate surface. Areas marked with red lines correspond to islands with a homogeneous structure.


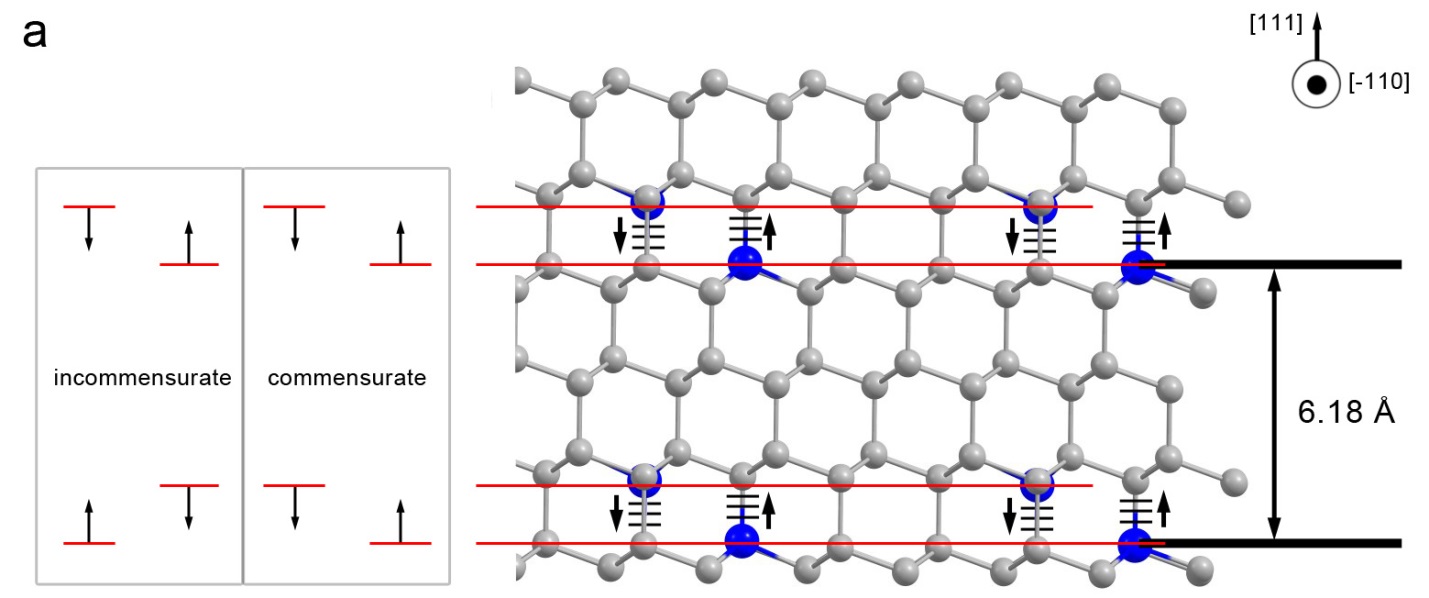


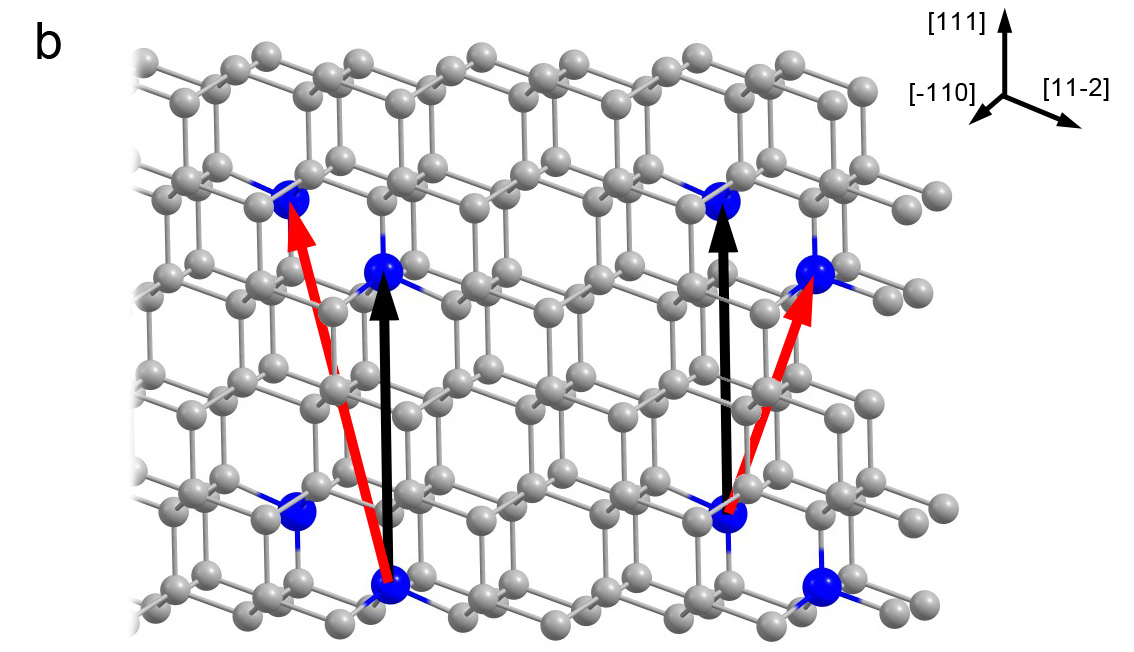


**Fig. S5 a** Diagram shows a distribution of the boron (blue) and carbon (gray) atoms in the (-110) plane. Arrows indicate the displacement of boron atoms along the [111] direction. The distance between boron atoms in the direction (111) is 6.18 Å which is equal to the length of biggest diagonal of the cubic diamond cell. On the left the crystallographic planes are shown. The distances between these planes are commensurate and incommensurate to that of in the host structure. **b Isometric illustration of the BDD structure. It shows directions of wave vectors whose length is incommensurate (red) and commensurate (black) with the vectors of the periodic host structure.**


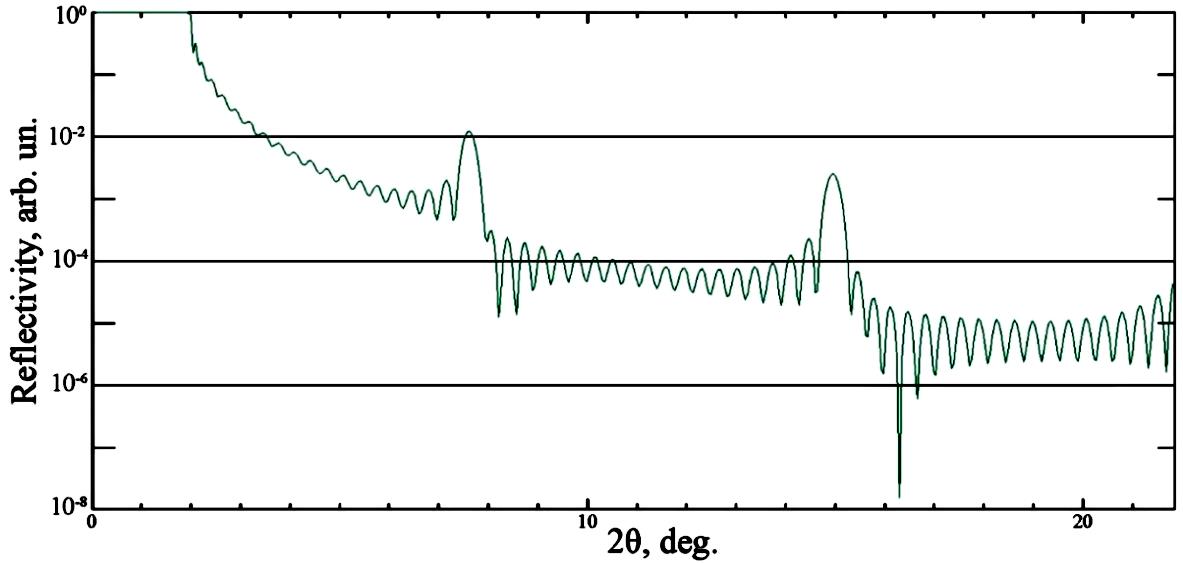


**Fig. S6.** The theoretical X-ray reflectivity curve obtained using IMD software.


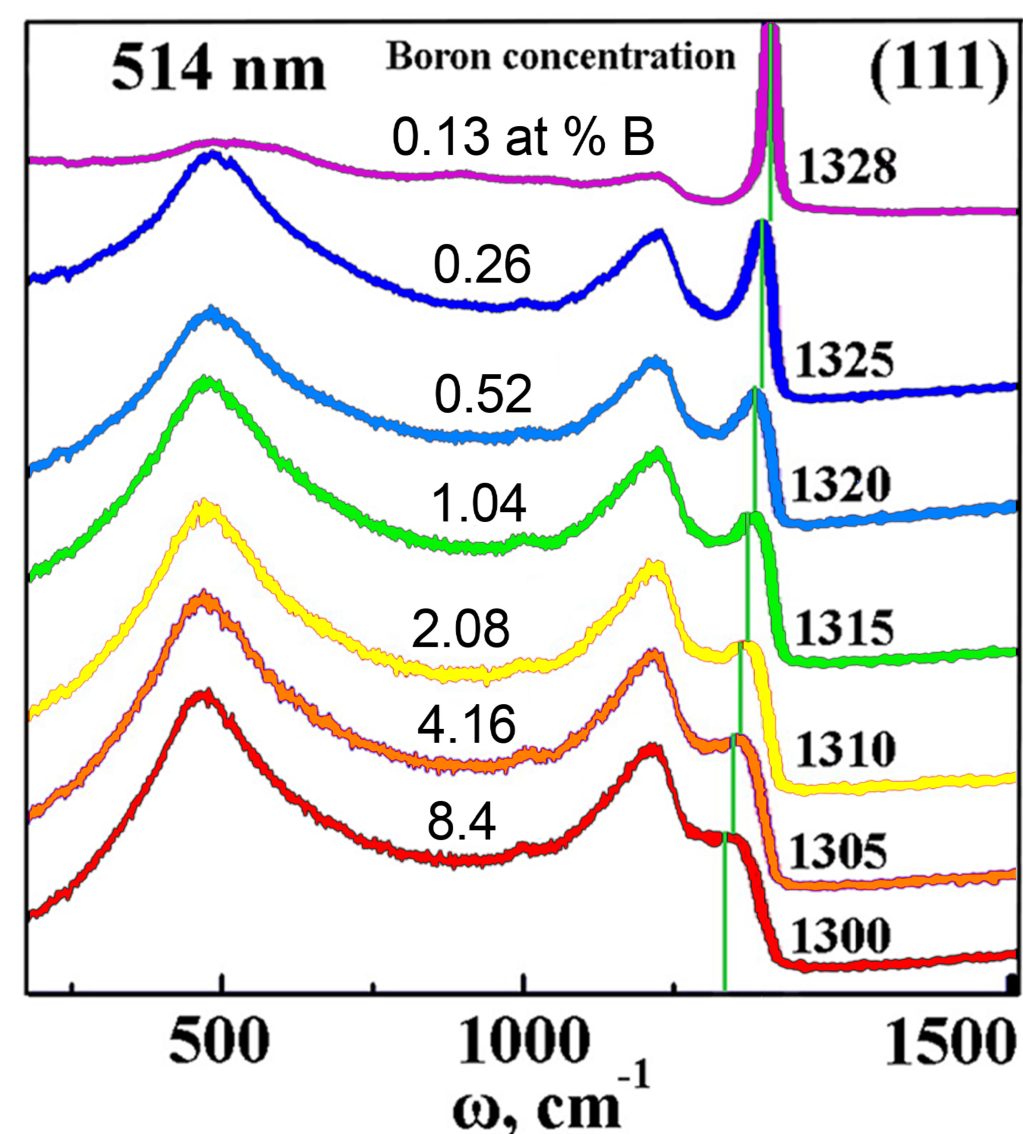


**Fig. S7.** Raman spectra from the as-grown and the opposite polished (upper spectrum) surfaces of BDD plate. The stepwise shifts of the phonon line depending on the boron concentration are marked by a green line. The horizontal axis scale from 1250 to 1500 cm^-1^ is expanded 2.5 times. The colors of the Raman spectra correspond to the colors of areas in Fig.7.

The boron concentrations were estimated according to the proposed structural model (see Additional file from [5]).

The Raman spectrum from the opposite surface (upper curve) reflects the vibrational properties of the BDD in the bulk at the boron concentration of 0.13 at % B (2×10^20^ cm^-3^). The intense 1328 cm^-1^ diamond phonon peak is the F_2g_ triply degenerated mode of O_h_ symmetry shifted by 4 cm^-1^ from the 1332 cm^-1^ peak in undoped diamond. The 588 and 1042 cm^-1^ Raman bands correspond to transverse (TA) and longitudinal (LA) acoustic phonon branches reflecting the phonon density states of BDD near the Brillouin zone boundary which are induced by the incorporation of boron into the substitution positions in the diamond lattice. The 890 cm^-1^ Raman band is assigned to the resonance-exited local mode which is activated by weakly bound boron atoms in diamond lattice. For the first time we assigned broad bands at 480 and 1230 cm^-1^ to the transverse acoustic (TA) and optical (TO) branches of the phonon density of states in atomically thin boron-carbon nanosheets and bilayers [5]. This assignment is based on the following experimental facts.

- These bands appear in Raman spectra of BDD with doping level of ~ 4×10^18^ cm^-3^ and their intensities increase linearly with increasing doping level from ~ 4×10^18^ up to ~ 2×10^20^ cm^-3^ (see Fig. 4a in Ref. 5).
- They have resonant character and are observed in Raman spectra excited with the 514-nm laser wavelength while they are absent in Raman spectra excited with the 257-nm laser wavelength (see Fig. 4b in Ref. 5).
- The intensities of the 588, 890, 1042 cm^-1^ defect-induced Raman bands observed in the BDD bulk do not change in the region of doping from ~4×10^18^ up to ~2×10^20^ cm^-3^. It means that boron atoms incorporate only in boron-carbon nanosheets and bilayers in this region of doping.
- The polarization properties of these new Raman bands indicate their D_6h_ symmetry (see Additional file 1.2 in [5]).
- The appearance of bands at 480 and 1230 cm^-1^ occurs simultaneously with a formation of the new shallow acceptor level at 37 meV and the observation of the 1*s* →n*s* electron-acceptor transitions in the electronic Raman spectra associated with boron-carbon nanosheets and bilayers.

The Raman spectra (lower curves) obtained by coarse mapping from islands on the (111) BDD surface depending on the boron concentration inside them are also shown. Raman spectra showed that the position of the diamond phonon peak varies stepwisely in the range from 1328 to 1300 cm^-1^ with a step of ~5 cm^-1^.


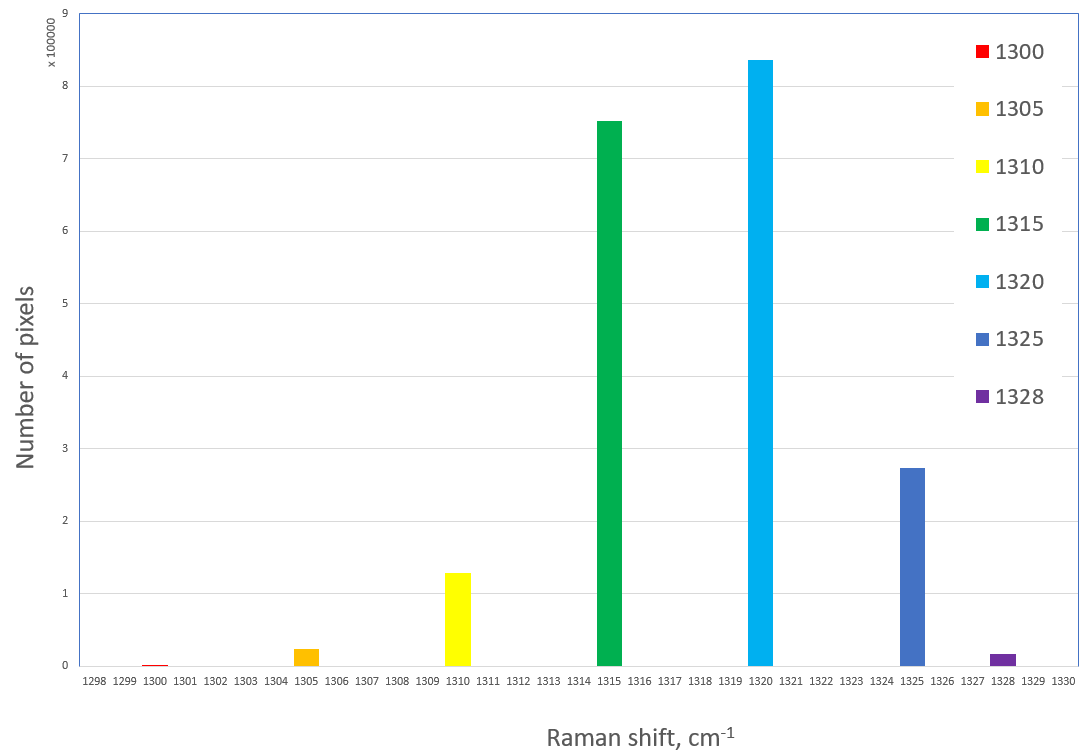


**Fig. S8.** Histogram providing the information about area ratio of islands with different boron concentrations (obtained from Fig.7).


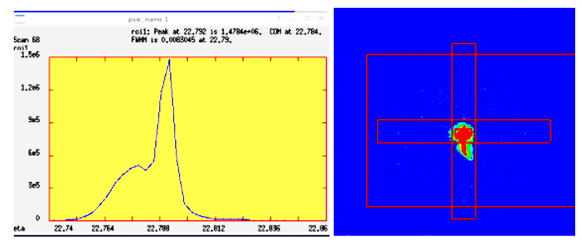


**Fig. S9**. The X-ray rocking curve (111) diamond reflection obtained with the synchrotron X-ray microbeam with energy of 7.8 keV demonstrates the reflection splitting caused by tensile stress.
